# SnO_2_ Nanoflower–Nanocrystalline Cellulose Composites as Anode Materials for Lithium-Ion Batteries

**DOI:** 10.3390/ma13143165

**Published:** 2020-07-15

**Authors:** Quang Nhat Tran, Il Tae Kim, Sangkwon Park, Hyung Wook Choi, Sang Joon Park

**Affiliations:** 1 Department of Chemical and Biological Engineering, Gachon University, Seongnam 13120, Korea; tran.nhat147@gmail.com (Q.N.T.); itkim@gachon.ac.kr (I.T.K.); 2 Department of Chemical and Biochemical Engineering, Dongguk University, Jung-gu, Seoul 04620, Korea; parksk@dongguk.edu; 3 Department of Electrical Engineering, Gachon University, Seongnam 13120, Korea; chw@gachon.ac.kr

**Keywords:** lithium-ion batteries, nanocrystalline cellulose, tin dioxide nanoflower, carbon-based conductive materials, CNC, SnO_2_

## Abstract

One of the biggest challenges in the commercialization of tin dioxide (SnO_2)_-based lithium-ion battery (LIB) electrodes is the volume expansion of SnO_2_ during the charge–discharge process. Additionally, the aggregation of SnO_2_ also deteriorates the performance of anode materials. In this study, we prepared SnO_2_ nanoflowers (NFs) using nanocrystalline cellulose (CNC) to improve the surface area, prevent the particle aggregation, and alleviate the change in volume of LIB anodes. Moreover, CNC served not only as the template for the synthesis of the SnO_2_ NFs but also as a conductive material, after annealing the SnO_2_ NFs at 800 °C to improve their electrochemical performance. The obtained CNC–SnO_2_NF composite was used as an active LIB electrode material and exhibited good cycling performance and a high initial reversible capacity of 891 mA h g^−1^, at a current density of 100 mA g^−1^. The composite anode could retain 30% of its initial capacity after 500 charge–discharge cycles.

## 1. Introduction

Over the past few years, metal oxides have attracted a great deal of attention as anode materials for rechargeable lithium-ion batteries (LIBs), owing to their high capacity, excellent rate capacity, and safety [1,2]. Among the various transition metal oxides used in LIBs, tin dioxide (SnO_2_) has been extensively investigated owing to its high theoretical capacity and unique properties. Furthermore, the synthesis procedures for SnO_2_ are cost-effective, facile, and achievable in environmentally benign conditions [3,4,5].

The following are the electrochemical reactions occurring in SnO_2_-based anodes:

SnO_2_ + 4Li^+^ + 4e^−^ = Sn + 2Li_2_O (1)

Sn + xLi + xe^−^ = Li_x_Sn (0 ≤ x ≤ 4.4). (2)

The highly reversible reaction in Equation (2) results in a theoretical capacity of 790 mA h g^−1^. However, nanosized SnO_2_-based LIB anodes have been demonstrated to exhibit higher capacities up to 1490 mA h g^−1^ [6,7,8]. Unfortunately, nanosized SnO_2_ anodes exhibit large volume changes (~300%) because of Li^+^ insertion–extraction [9,10,11,12]. Furthermore, most metallic, magnetic nanoparticles are prone to severe aggregation because of their high specific surface area and the interaction between magnetic dipoles, which degrades the performance of anode materials [13].

Various methods have been used to develop SnO_2_ nanostructures and improve their electrochemical performance. SnO_2_ nanoflowers (SnO_2_ NFs) exhibit the best electrochemical performance among all of the SnO_2_ nanostructures investigated to date for LIB anode applications [14,15,16,17]. The various advantages offered by SnO_2_NF LIB electrodes include a large surface area, a unique and stable morphology, and fast ion and electron transfer characteristics. Moreover, SnO_2_ has been combined with carbon-based materials to form composite materials to alleviate the change in volume, to create good electrical contact, and to increase the number of electronic transport pathways [18,19,20,21].

Nanocrystalline cellulose (CNC), which is synthesized by the sulfuric acid hydrolysis of cellulose nanofiber, or native cellulose, has a nanoscale dimension with a diameter of 5–30 nm and reactive surface properties. It is thought to be a more efficient material when incorporated in LIBs because of the nanoscale of many of the active materials, leading to a high specific surface area [22,23,24]. Nanocrystalline cellulose not only has many advantages, such as being low-cost, lightweight, and having physicochemical robustness, but has also exhibited an excellent colloidal stability. Thus, it shows great potential for use in nanocellulose-based materials, and in many types of LIBs, such as electrodes, electrolytes, and separators. In addition, nanocrystalline cellulose can be dispersed uniformly in solutions, exhibits negative surface charges, and is used as a template and dispersant for the synthesis of nanomaterials to prevent the aggregation of metal oxide nanoparticles [25]. Moreover, the pyrolysis of CNC occurring during the annealing of nanoparticles renders it highly conductive, leading to an improvement in the electrochemical performance of the LIB electrode materials [21].

In this study, we prepared CNC–SnO_2_ NF composites, with the merits of both SnO_2_ and pyrolyzed CNC. CNC acted as a carrier and dispersant, for preventing the aggregation of the SnO_2_ NFs, and to alleviate the change in volume of SnO_2_. The prepared composites were used as LIB anodes.

## 2. Experiment Details

### 2.1. Materials

The CNC suspension used in this study was obtained from SK innovation (Daejeon, Korea). Tin (II) chloride dihydrate (SnCl_2_·2H_2_O) and sodium citrate dihydrate (C_6_H_5_Na_3_O_7_·2H_2_O) were purchased from Sigma-Aldrich reagent Co. Ltd. (St. Louis, MO, USA).

### 2.2. Preparation of CNC–SnO_2_NF Composites

First, certain amounts of SnCl_2_·2H_2_O (0.1128 g) and C_6_H_5_Na_3_O_7_·2H_2_O (0.2941 g) were dissolved in an 80 mL ethanol–deionized (DI) water solution. The resulting suspension was stirred continuously for 1 h. Then, 0.1 g CNC was added to the mixture, which was vigorously stirred for 0.5 h, and the resulting solution was then transferred to a 100 mL stainless steel autoclave. After heating at 180 °C for 8 h, the autoclave was naturally cooled to room temperature. The obtained sample was washed with DI water by centrifugation at 8000 rpm for 10 min. The powder samples were then annealed at 500 and 800 °C for 2 h under a nitrogen atmosphere. The final composite products obtained after annealing at 500 and 800 °C were labeled as CNC–SnO_2_NF500 and CNC–SnO_2_NF800, respectively.

### 2.3. Material Characterization

The structures and morphologies of the obtained composite materials were observed using scanning electron microscopy (SEM, S-4700, Hitachi Ltd., Tokyo, Japan). Transmission electron microscopy (TEM) analysis was carried out on a FEI Tecnai F30S-Twin transmission electron microscope, (Tecnai, F30S-Twin, Hillsboro, OR, USA). The X-ray diffraction (XRD) analysis of the obtained composite materials was carried out on a diffractometer (Rigaku/Smartlab, Tokyo, Japan) (40 kV, 30 mA X-Ray generator) at the Smart Materials Research Center for IoT, at Gachon University. The XRD patterns of the composites were recorded over the 2θ range of 10°–80° at a scanning rate of 1.0° min^−1^. The diffractometer was equipped with a Kβ filter for Cu. The Raman spectra of the composites were recorded on a micro-Raman instrument (Monora500i, ANDOR, Belfast, Northern Ireland) equipped with a 533 nm He-Ne laser (12 mW). Thermogravimetric analysis (TGA) was conducted in air at a heating rate of 10 °C min^−1^.

### 2.4. Electrochemical Characterization

Electrochemical tests were carried out with CR2032-type coin cells. The working electrodes (mass load 0.088 mg/cm^2^) were prepared by compressing slurries comprising 70 wt % active materials, 15 wt % Super P, 15 wt % polyvinylidene fluoride, and N-methyl pyrrolidone onto a copper foil (r = 0.6 cm). The electrodes were dried at 70 °C for 24 h in a vacuum oven and the cells were assembled in an argon-filled glove box. A polyethylene membrane and lithium foil were used as the separator and counter electrode, respectively. A solution of 1 M LiPF_6_ in ethylene carbonate/diethylene carbonate (1:1 in volume) was used as the electrolyte. Charge and discharge experiments were carried out over the potential range of 0.01–2.0 V (vs Li/Li^+^) at a constant current density of 100 mA g^−1^ with a battery cycler (WBCS3000, WonAtech, Seoul, Korea) system.

## 3. Results and Discussion

**Figure 1:**
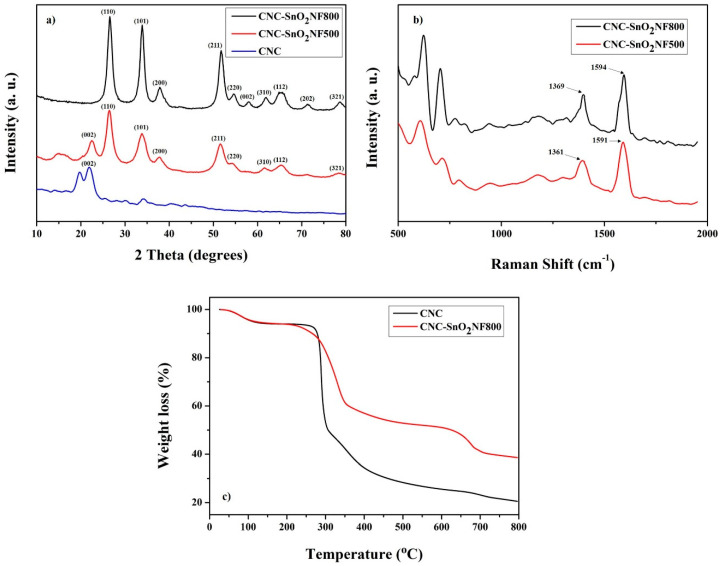
(**a**) XRD patterns of nanocrystalline cellulose (CNC) and the CNC–tin dioxide (SnO_2_) nanoflower (NF) composites, (**b**) Raman spectrum of the CNC–SnO_2_NF composite, and (**c**) TGA profiles of the CNC and CNC–SnO_2_NF800 nanocomposites in an air atmosphere with the heating rate of 10 °C min^−1^.

Figure 1a shows the XRD patterns of CNC and the CNC–SnO_2_NF composites. The CNC–SnO_2_NF500 and CNC–SnO_2_NF800 composites showed peaks characteristic of SnO_2_ along with graphitic peaks. In the case of CNC, the peaks at 2θ = 19.8° and 22.6° could be indexed to ICSD data, PDF #03-0289 (native cellulose) and PDF #03-0228 (cellulose), respectively, and in agreement with the report from SK innovation. The presence of SnO_2_ in the CNC–SnO_2_NF composites was confirmed by the peaks observed at around 2θ = 26.8°, 34.1°, 38.1°, and 51.8° corresponding to the (110), (101), (200), and (211) planes of SnO_2_, respectively (ICSD PDF 21-1250). Additionally, the peak corresponding to the (002) plane of CNC, which was observed in the XRD patterns of the CNC and CNC–SnO_2_NF500 samples, could not be observed in the XRD pattern of the CNC–SnO_2_NF800 composite. This indicates that CNC was successfully pyrolyzed [21,26,27], which is further confirmed by the Raman spectrum, as shown below. In the case of the composite materials, the graphitic carbon (002) peak overlapped the (110) peak of SnO_2_. Moreover, the (110), (101), (200), and (211) peaks shifted toward the right and became more intense and sharper in the case of CNC–SnO_2_NF800, indicating the high purity and crystallization of the composite.

To further examine the structure of the CNC–SnO_2_NF composites, their Raman spectrum was recorded over the wavenumber range of 500–2000 cm^−1^ (Figure 1b). CNC–SnO_2_NF800 composites showed the D-band peak at ~1369 cm^−1^ and the G-band peak at ~1594 cm^−1^, while CNC–SnO_2_NF500 exhibited the D-band peak at ~1361 cm^−1^ and the G-band peak at ~1591 cm^−1^, corresponding to the disordered and graphitic carbon atoms, respectively. These indicated the presence of the carbon formed by the pyrolysis of CNC in the CNC–SnO_2_NF composite and were consistent with previous reports [20,28,29]. The composite showed sharp D and G-band peaks because of the high carbonization temperature. Moreover, the sharpness of the D and G-band peaks and the I_G_/I_D_ intensity ratio increased with an increase in the pyrolysis temperature. This was consistent with the results reported previously [30,31]. In addition, the peaks observed at around 600–750 cm^−1^ corresponded to the Sn-O bonds in the SnO_2_ NFs [32]. This further confirmed the presence of SnO_2_ NFs in the composites. In order to quantitatively determine the carbon content for CNC and CNC–SnO_2_NF800, thermogravimetric analysis (TGA) was implemented and Figure 1c shows the TGA profiles of the CNC and CNC–SnO_2_NF800 nanocomposites. The CNC exhibited weight loss mainly in the temperature range of 250–400 °C, and for CNC–SnO_2_NF800, the weight loss between 400 and 750 °C corresponded to pyrolysis of the CNC. The final residual weight of 38% was obtained for the final SnO_2_ NFs and pyrolyzed CNC. Therefore, the mass percentage of the final pyrolyzed CNC can be calculated at 21% in the electrode.

**Figure 2:**
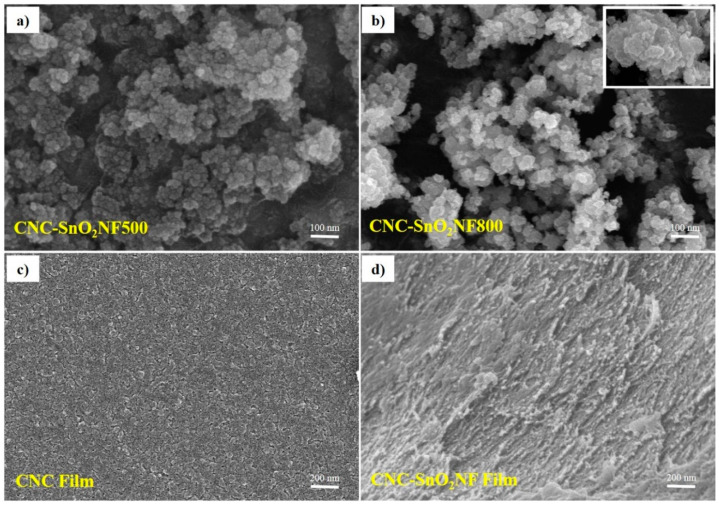
SEM images of (**a**) CNC–SnO_2_NF500, (**b**) CNC–SnO_2_NF800, (**c**) CNC, and (**d**) CNC–SnO_2_NF Film.

The nanostructures and morphologies of the CNC–SnO_2_NF composites were examined using SEM and TEM. The SEM images of CNC–SnO_2_NF500, CNC–SnO_2_NF800, CNC, and CNC–SnO_2_NF are shown in Figure 2a–d, respectively. As can be observed from Figure 2a,b, both the CNC–SnO_2_NF500 and CNC–SnO_2_NF800 composites showed a uniform flower-like structure consisting of SnO_2_ nanosheets. However, the composite annealed at 800 °C showed more nanosheets and aggregation than that annealed at 500 °C. The magnified image shown in the inset of Figure 2b shows the highly porous surface and flower-like structure of the CNC–SnO_2_NF800 composite. Figure 2c shows a typical SEM image of the CNC film with a heterogeneous surface. In the case of CNC–SnO_2_NF, the SnO_2_ NFs were uniformly distributed on the surface of CNC (Figure 2d). Compared with the CNC film, the CNC–SnO_2_NF film showed a porous surface, and hence showed improved electrode performance.

**Figure 3:**
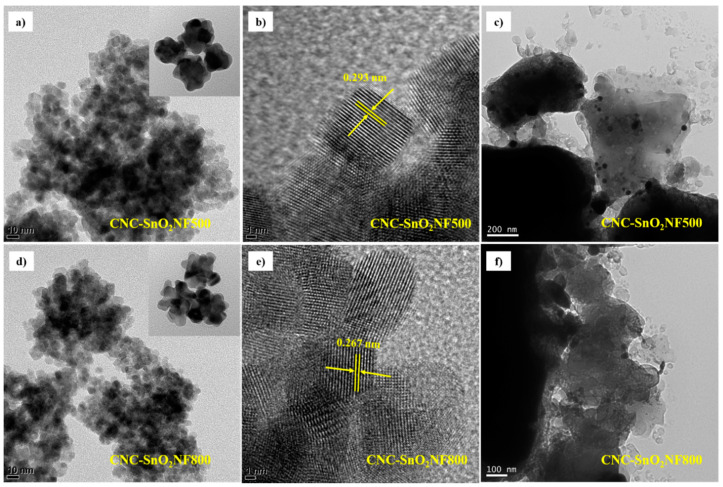
(**a**,**d**) Typical TEM, (**b**,**e**) HRTEM, (**c**,**f**) and low-magnification TEM images of CNC–SnO_2_NF500 and CNC–SnO_2_NF800.

The structure of the CNC–SnO_2_NF was further examined using TEM and high-resolution TEM (HRTEM), as shown in Figure 3a–f. Both the CNC–SnO_2_NF500 and CNC–SnO_2_NF800 composites showed uniformly distributed SnO_2_ NFs, as can be observed from Figure 3a,d, respectively. The SnO_2_ NFs were highly crystalline, with an average particle size of 8–10 nm. Moreover, the magnified HRTEM images in Figure 3b,e show the lattice fringes of the CNC–SnO_2_NF500 and CNC–SnO_2_NF800 composites, respectively. As can be observed from Figure 3b, the lattice parameter of the CNC–SnO_2_NF500 composite was 0.293 nm, while that of the CNC–SnO_2_NF800 composite was 0.267 nm (Figure 3e), corresponding to the (110) and (101) lattice planes of SnO_2_ nanoparticles. These results indicate that the CNC–SnO_2_NF800 nanocomposite showed better uniformity and crystallization and had a smaller nanosheet size than the CNC-SnO_2_NF500 composite. This is consistent with the XRD results. In addition, Figure 3c,f shows the low-magnification TEM images of the CNC–SnO_2_NF500 and CNC–SnO_2_NF800 composites, respectively. Both of the images reveal that the SnO_2_ NFs were uniformly distributed in the CNC layers, and the CNC–SnO_2_ composites showed SnO_2_ NF aggregation. These results are consistent with the SEM results. These results indicate that the SnO_2_ NFs were successfully coated onto the CNC layer, and improved the electrochemical performance of the composite anodes.

**Figure 4:**
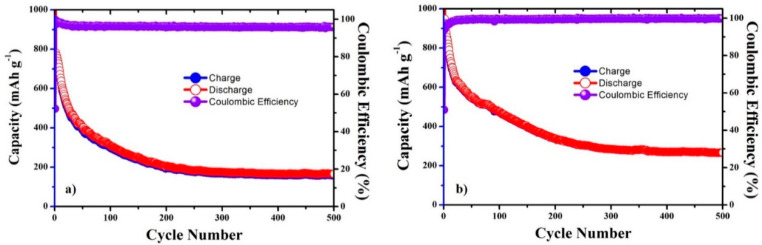
Cyclic performances of the (**a**) CNC–SnO_2_NF500 and (**b**) CNC–SnO_2_NF800 composite electrodes at 100 mA g^−1^.

The CNC–SnO_2_NF (CNC–SnO_2_NF500 and CNC–SnO_2_NF800) composites were used as LIB anodes. The charge–discharge capacity curves of the composite anodes, at a current density of 100 mA g^−1^, are shown in Figure 4a,b. SnO_2_ and CNC exhibited theoretical capacities of 1490 mA h g^−1^ and 360 mA h g^−1^, respectively. Therefore, the CNC–SnO_2_NF500 and CNC–SnO_2_NF800 composites showed different charge–discharge capacities because of the differences in their annealing temperatures and structures. Figure 4a shows the cycle performance properties of the CNC–SnO_2_NF500 composite electrode. The initial discharge capacity, charge capacity, and initial Coulomb efficiency (ICE) of the electrode were 1391 mA h g^−1^, 726 mA h g^−1^, and 52.21%, respectively. On the other hand, the initial discharge capacity, charge capacity, and ICE of the CNC–SnO_2_NF800 composite electrode were 1752 mA h g^−1^, 891 mA h g^−1^, and 50.84%, respectively (Figure 4b). The first discharge capacities of both of the electrodes were higher than the theoretical capacities of SnO_2_ and CNC for the following reasons: (1) the formation of solid–electrolyte interface (SEI) layers on the surface and the decomposition of electrolyte during the first discharge process, leading to an increase in the irreversible capacity of the electrodes; (2) the carbon content of nanocellulose after pyrolysis in nanocomposites; (3) tiny vacancies existing between the SnO_2_ and nanocellulose that can buffer the SnO_2_ volume expansion, which could be seen in the SEM and HRTEM results; and (4) the constitution of a space charge layer comprised of lithium ions at the interface between the lithium salt and metal particles [7,28,33,34]. The initial Coulombic efficiencies of the nanocomposites were 52.21% and 50.84% for CNC–SnO_2_NF500 and CNC–SnO_2_NF800 composites, respectively, which were very close to the theoretical values. The composite annealed at 800 °C exhibited a higher initial discharge capacity (1752/1391 mA h g^−1^) and charge capacity (891/726 mA h g^−1^) than that annealed at 500 °C. These results are consistent with the XRD and TEM results. Furthermore, the CNC–SnO_2_NF500 and CNC–SnO_2_NF800 composites showed similar cycling performance and Coulombic efficiency after 500 cycles. The capacities of both of the electrodes decreased rapidly in the first 50 cycles, decreased slightly in the next 150 cycles, and remained constant after 200 cycles. The electrodes could retain 30% of their initial capacity after 500 cycles. This can be attributed to the collapse of the SnO_2_ NF structure because of the volume change during the charge–discharge processes, which further decreased the reversible capacity of the electrode. Compared with the CNC–SnO_2_NF500 electrode, the CNC–SnO_2_NF800 electrode exhibited improved cyclic stability and electrochemical performance. After 500 cycles, the reversible capacity of the CNC–SnO_2_NF800 electrode was 267 mA h g^−1^, which is higher than that of the CNC–SnO_2_NF500 composite (158 mA h g^−1^). This is consistent with previously reported results [21], according to which the performance of CNC–SnO_2_-based LIB anodes improves with an increase in the CNC pyrolysis temperature, and the conductivity of CNC can increase the capacity of CNC–SnO_2_ composite materials.

**Figure 5:**
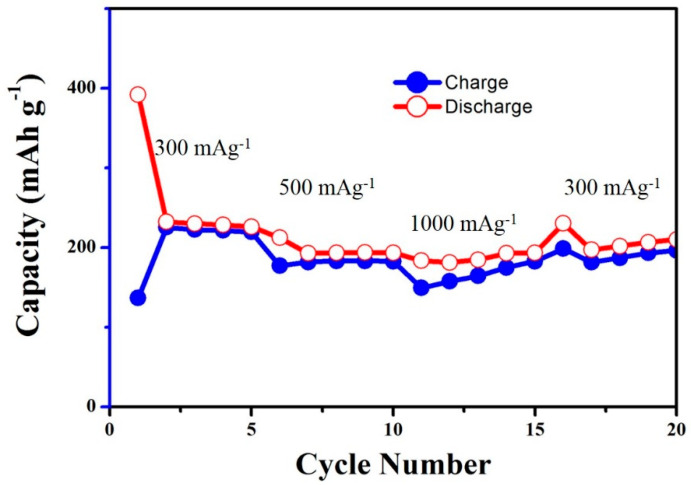
The rate performance at different current densities of CNC–SnO_2_NF800 composite electrodes between 0.01 and 2 V. The rate performance experiment was carried out by using the lithium-ion battery (LIB)’s cell after 500 cycles at a current density of 100 mA g^−1^.

The rate capability and cyclability of the composite, with the discharge–charge capacity at different current densities from 300 to 1000 mA g^−1^, was obtained for the CNC–SnO_2_NF800 sample, and the results are shown in Figure 5. The rate performance experiment was carried out by using the LIB cell after 500 cycles at a current density of 100 mA g^−1^. The average reversible discharge capacities at 300, 500, and 1000 mA g^−1^ for the CNC–SnO_2_NF800 nanocomposites were 261.9, 197.2, and 187.3 mA h g^−1^, respectively. When the specific current was retained at 300 mA g^−1^, a capacity of 209.4 mA h g^−1^ was achieved, demonstrating good rate performance. Comparatively, with CNC–SnO_2_NF800 nanocomposites, at a discharge capacity of 267.1 mA h g^−1^, a current density of 100 mA g^−1^, and after 500 cycles, the composite material showed stable cycling properties at different current densities.

**Figure 6:**
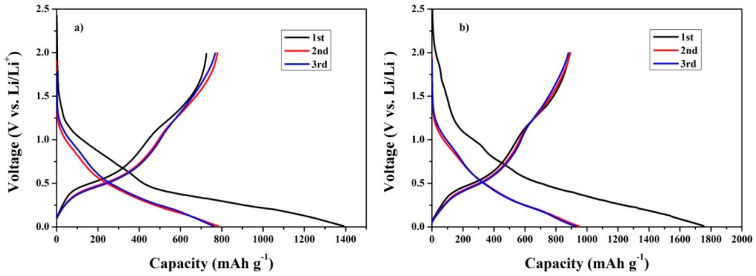
Initial voltage profiles of the (**a**) CNC–SnO_2_NF500 and (**b**) CNC–SnO_2_NF800 composite electrodes at 100 mA g^−1^.

Figure 6a,b show the typical charge and discharge capacities of the CNC–SnO_2_NF500 and CNC–SnO_2_NF800 composite electrodes during the first, second, and third cycles over the potential range of 0.01–2.5 V (Li/Li^+^), at a current density of 100 mA g^−1^. The CNC–SnO_2_NF500 electrode showed a first discharge–charge capacity of 1391/726 mA h g^−1^ with a low ICE of 52% because of the decomposition of the electrolyte and the generation of the SEI layer. The CNC–SnO_2_NF800 composite showed a first discharge–charge capacity of 1752/891 mA h g^−1^, corresponding to an initial ICE of 50%. The electrodes exhibited a large irreversible capacity loss during the first cycle owing to the material properties of SnO_2_. This is a common phenomenon in LIBs [1,2,3,35]. Furthermore, the discharge–charge curves changed slightly with an increase in the number of cycles. The curve shape remained the same during the second and third cycles, indicating that the composite electrodes exhibited stable cyclic performance from the second cycle onwards.

**Figure 7:**
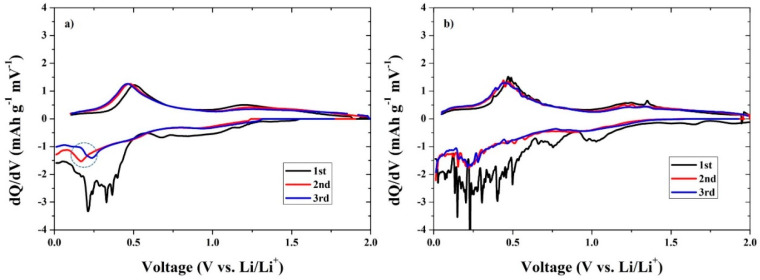
Differential capacity plots of the (**a**) CNC–SnO_2_NF500 and (**b**) CNC–SnO_2_NF800 composite electrodes over the potential range of 0.01–2.00 V.

Figure 7 shows the differential capacity plots of the CNC–SnO_2_NF500 and CNC–SnO_2_NF800 composite electrodes for the first three cycles over the potential range of 0.001–2.00 V. The differential capacities of the electrodes in the second and third cycles were significantly different from those in the first cycle. The curves indicated the occurrence of typical oxidation and reduction electrochemical processes. The first-cycle curve of CNC–SnO_2_NF500 (Figure 7a) showed two reduction peaks at 0.51 and 1.2 V, attributable to the Sn oxidation (SnO_2_ + 4Li^+^ + 4e^−^ = Sn + 2Li_2_O) process. The reduction peaks at around 0.2, 0.3–0.4, and 0.7 V can be ascribed to the lithiation (Li_x_Sn) of Sn (Sn + xLi + xe^−^ = Li_x_Sn (0 ≤ x ≤ 4.4)). Over the potential range of 0.001–2.0 V, the CNC–SnO_2_NF800 (Figure 7b) and CNC–SnO_2_NF500 electrodes showed similar differential capacity curves. The first-cycle differential capacity curves of the CNC–SnO_2_NF800 and CNC–SnO_2_NF500 electrodes showed clear redox peaks at (0.2 V, 0.5 V) and (0.75 V, 1.32 V). The first redox pair corresponds to the alloy–dealloy process (Li_x_Sn → Sn + xLi^+^ + xe^−^) and the formation of the SEI films. The other pair can be attributed to the formation of SnOx by the conversion reaction (Sn/SnO + Li_2_O → SnO/SnO_2_ + 2Li^+^ + 2e^−^). Moreover, the differential capacity curves of the second and third cycles overlapped in the case of CNC–SnO_2_NF800 electrodes (Figure 7b), indicating the stability of its reversible capacities. However, a shift on the discharge peak is clearly observed in Figure 7a during the second and third cycles, which revealed unstable reversibility of the electrochemical reactions in the case of the CNC–SnO_2_NF500 electrode. These results are also consistent with the initial voltage profiles and cyclic performances.

**Figure 8:**
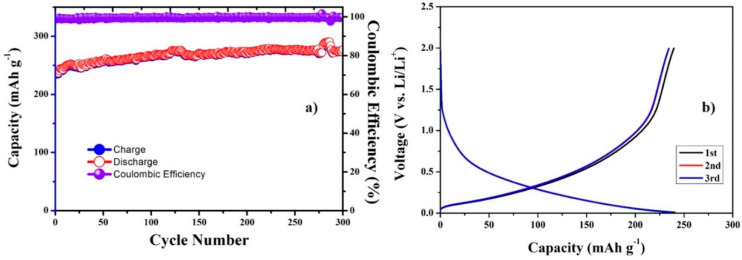
(**a**) Cyclic performance and (**b**) initial voltage profiles of the pyrolyzed CNC electrode at 100 mA g^−1^. The pyrolysis temperature is 800 °C.

For comparison, the electrochemical properties of CNC film pyrolyzed at 800 °C were investigated. The cycling performance and initial voltage profiles of the pyrolyzed CNC are shown in Figure 8. Figure 8a shows the cycle performance of the pyrolyzed CNC electrode for 300 cycles. The CNC–SnO_2_NF800 composite (Figure 4b and Figure 6b) showed significantly improved initial discharge (1752/237 mA h g^−1^) and charge capacities (891/234 mA h g^−1^) during 300 cycles, as compared with the pyrolyzed CNC film. After 200 cycles, the CNC-SnO_2_NF800 electrode showed a higher reversible capacity (339 mA h g^−1^) than the pyrolyzed CNC electrode (274 mA h g^−1^). However, the capacity of the pyrolyzed CNC electrode (Figure 8a) showed an increasing trend, and remained stable during 300 cycles, while that of the CNC–SnO_2_NF800 electrode decreased rapidly during the first 100 cycles and showed a capacity of 284 mA h g^−1^, similar to that of the pyrolyzed CNC electrode after 300 cycles (276 mA h g^−1^). In addition, the pyrolyzed CNC electrode’s capacity loss after the first cycle at 100 mA g^−1^ (Figure 8b) was lower than that of the CNC–SnO_2_NF800 composite electrode (Figure 6b). These results suggest that SnO_2_ cannot maintain the material structure of SnO_2_-based LIB electrodes because of its volume change during the charging–discharging processes. In addition, in the cycling process, the capacity of SnO_2_-based anode material starts to drop rapidly and has almost no capacity after 50 cycles, as shown in many previous reports [20,35,36,37]. Thus, the excellent performance of the CNC–SnO_2_NF500 and CNC–SnO_2_NF800 composite electrodes can only be attributed to the presence of pyrolyzed CNC.

**Figure 9:**
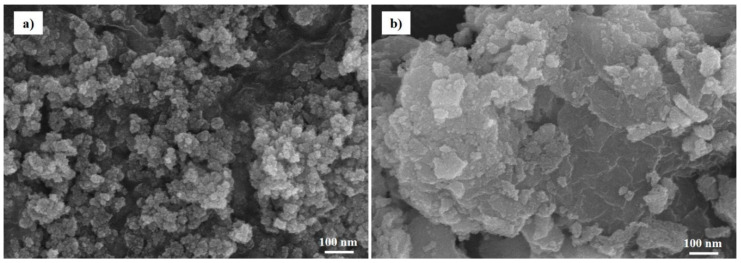
SEM images of the CNC–SnO_2_NF800 electrode (**a**) before and (**b**) after 200 cycles.

To further confirm the structural stability of the CNC–SnO_2_NF800 composite, SEM images of it after 200 cycles were obtained (Figure 9). The SEM images of the electrode before and after cycling (200 cycles) are shown in Figure 9a,b, respectively. It can be observed from the images that the electrode could not maintain the SnO_2_ NF structure, and showed particle aggregation after 200 cycles. However, the SnO_2_ NFs did not collapse completely, and the material network structure could be observed even after cycling. SnO_2_ NFs often tended to be highly aggregated because of their high surface energy and magnetic dipole interaction. Thus, CNC can cover and separate SnO_2_ NFs, in combination with the dispersion effect, to reduce the aggregation. The negatively charged surface of CNC can also improve the dispersion and colloidal stability of nanoparticles during cycling, which can be clearly observed from the SEM and TEM images. Therefore, the CNC-incorporated conductive network, which prevented the aggregation and transformation of the SnO_2_ NFs during the charge–discharge processes, should be further optimized.

## 4. Conclusions

In summary, we successfully prepared CNC–SnO_2_NF composites, which showed excellent electrochemical performance as LIB electrodes. CNC not only prevented the agglomeration and change in volume of the SnO_2_ NFs, but also served as a conducting base after high-temperature annealing of the SnO_2_ NFs. Moreover, the CNC structure enhanced the discharge–charge capacity and cycling performance of the SnO_2_ NFs during the cycling process. In addition, the CNC–SnO_2_NF800 composite showed an initial discharge capacity of 1752 mA h g ^−1^, and maintained a stable discharge capacity of 267 mA h g^−1^ after 500 cycles at 100 mA g^−1^. The low-cost, lightweight, flexible, and environmentally favorable properties of CNC enable it to become an ideal green, non-toxic, and effective protectant, or dispersant matrix, in the development of metal oxide–nanocellulose composites, possibly applicable to advanced electrodes for LIBs. In particular, the properties of CNC may be utilized for a freestanding electrode in the future.
